# Functional and histologic imaging of urinary bladder wall after exposure to psychological stress and protamine sulfate

**DOI:** 10.1038/s41598-021-98504-9

**Published:** 2021-09-30

**Authors:** Tetsuichi Saito, T. Kevin Hitchens, Lesley M. Foley, Nishant Singh, Shinsuke Mizoguchi, Masahiro Kurobe, Daisuke Gotoh, Teruyuki Ogawa, Tomonori Minagawa, Osamu Ishizuka, Christopher Chermansky, Jonathan Kaufman, Naoki Yoshimura, Pradeep Tyagi

**Affiliations:** 1 Department of Urology, School of Medicine, University of Pittsburgh, E313 Montefiore Hospital, 3459 Fifth Avenue, Pittsburgh, PA USA; 2 Animal Imaging Center, University of Pittsburgh, Pittsburgh, USA; 3 Department of Neurobiology, University of Pittsburgh, Pittsburgh, USA; 4 Department of Urology, Shinshu University, Matsumoto, Japan; 5 Lipella Pharmaceuticals Inc, Pittsburgh, PA USA

**Keywords:** Physiology, Medical research, Urology

## Abstract

To quantify the urinary bladder wall T_1_ relaxation time (T_1_) before and after the instillation contrast mixture in rats previously subjected to water avoidance stress (WAS) and/or acute exposure to protamine sulfate (PS). Female Wistar rats were randomized to receive either sham (control) or 1 h of WAS for ten consecutive days before the evaluation of nocturnal urination pattern in metabolic cages. T_1_ mapping of urinary bladder wall at 9.4 T was performed pre- and post- instillation of 4 mM Gadobutrol in a mixture with 5 mM Ferumoxytol. Subsequently, either T_1_ mapping was repeated after brief intravesical PS exposure or the animals were sacrificed for histology and analyzing the mucosal levels of mRNA. Compared to the control group, WAS exposure decreased the single void urine volume and shortened the post-contrast T_1_ relaxation time of mucosa- used to compute relatively higher ingress of instilled Gadobutrol. Compromised permeability in WAS group was corroborated by the urothelial denudation, edema and ZO-1 downregulation. PS exposure doubled the baseline ingress of Gadobutrol in both groups. These findings confirm that psychological stress compromises the paracellular permeability of bladder mucosa and its non-invasive assay with MRI was validated by PS exposure.

## Introduction

Bladder Pain Syndrome/Interstitial Cystitis (BPS/IC) is a debilitating condition associated with severe bladder pain and lower urinary tract symptoms (LUTS). Importantly, disease history of patients afflicted with LUTS, functional pain disorders, and BPS/IC^1–5^ tends to show a prior or recent exposure to psychological stress, but whether stress is coincident, or a causative factor of BPS/IC is yet to be investigated.

The epidemiologic link between the psychological stress and the BPS/IC symptoms of urinary bladder pain and increased urinary frequency have received mechanistic support from the evidence of visceral hyperalgesia in rodent models of psychological stress^6–9^. Moreover, a structurally deficient urothelium of BPS/IC patients with the proliferation of mast cells^4,5,10–12^ being mirrored in the rodent models of chronic water avoidance stress (WAS)^13,14^ supports their validity as a rodent model for BPS/IC. However, the mechanistic link between psychological stress and the BPS/IC symptoms remains to be tested in a radiation-free and in a clinically viable manner^15,16^ and consequently there is an unmet need for a minimally invasive assay of mucosal permeability without the site-selection bias of bladder biopsy^2^. Such a permeability assay could have many clinical applications including objective phenotyping of the heterogeneity in BPS/IC symptoms for informing the treatment selection and the development of new drugs. Instead of measuring the concentration of instilled polar dyes, *i.e.,* Evans blue or Trypan blue in bladder harvested after animal sacrifice to measure the bladder permeability^17–20^, here we seek to non-invasively measure the bladder permeability in a clinically viable manner by replacing the instillation of polar dyes with the instillation of an extracellular T_1_ shortening agent, Gadolinium chelate^21^ and then compute the concentration of instilled Gadolinium chelate diffusing into bladder mucosa from the changes in the physical parameter, proton spin–lattice relaxation time (T_1_ relaxation time) of mucosa measured via Magnetic Resonance Imaging (MRI), pre and post-instillation of the Gadolinium chelate- Gadobutrol. Gadobutrol being a paramagnetic agent develops a magnetic moment in the magnetic field of scanner and the resulting induction of a local magnetic field shortens the T_1_ relaxation time of water protons present in tissue to brighten the tissue being imaged. The premise for the proposed translational research is most strongly supported by the successful use of MRI for measuring the blood brain barrier permeability compromised by inflammation in mildly demented patients^22^. Since the volume of distribution for the instilled or injected Gadolinium chelate is restricted to the vascular and extravascular-extracellular space available in a given tissue^23^; edematous changes in bladder wall provoked by WAS^13,14^ are expected to enlarge the physical space for the residence of diffused Gadobutrol and affect the MR images of diseased bladder, accordingly.

Therefore, considering that compromised mucosal permeability alters the biochemical milieu of bladder wall^17^, we hypothesize that the measurement of the physical parameter, T_1_ relaxation time in milliseconds (ms) for bladder mucosa measured pre and post-instillation of Gadobutrol will serve as an index for mucosal permeability in WAS model. Here, we quantified the T_1_ relaxation time of bladder wall- the first order rate constant for the exponential rise in signal intensity from a series of T_1_ weighted spin echo images acquired at Variable Repetition Time (VTR)- a method previously used on human bladder at 1.5T^24^. Therefore, instead of a biochemical assay for permeability in BPS/IC patients^2,10,16^, the overall focus of this research study is to empirically demonstrate the utility of contrast enhanced MRI for assaying the mucosal permeability of WAS rat with or without the intravesical exposure to protamine sulfate (PS)—an agent known to increase mucosal permeability of rodent^25–27^ as well as of human bladder^16^.

## Methods

All experiments were conducted on ten-week-old female Wistar rats (n = 21) in accordance with the National Institutes of Health Guide for the Care and Use of Laboratory Animals and ARRIVE guidelines and approved by the University of Pittsburgh Institutional Animal Care and Use Committee.

### Water Avoidance Stress (WAS) model

Animals were randomly divided into control (N = 9) or WAS (N = 12) groups. Animals underwent 1-h exposure to WAS or control setting for 10 consecutive days as published previously^28^. Briefly, rats from both groups were placed on a glass platform (8 × 8 cm) in the middle of a plastic container, 90 cm in diameter and 50 cm in height, which was left empty for the control group and filled with 49 cm of water for the WAS group. Since psychological stress is known to modulate colonic motility, number of fecal pellets excreted after WAS were measured.

### Metabolic cage

After 10 days of WAS, all rats were placed in a metabolic cage (Tecniplast, Buguggiate VA, Italy) for 12 h (from 7 pm to 7am) with food and water ad libitum. Voiding frequency, single void urine volume and total urine volume were measured.

### Voxel-wise T_1_ mapping

As described recently for mouse bladder^29^, MRI was performed in a 9.4 T/30 cm Bruker AVANCE III HD scanner (Bruker BioSpin, Billerica, MA, USA) running ParaVision 6.0.1 equipped with a 12 cm BGA-12SHP gradient set, an 86 mm transmit coil and 35 mm 2 × 2 receiver array over the bladder. Rats were maintained under isoflurane anesthesia via a nose cone (2–3% in 1:1 Oxygen: Air). Following pilot scans, a Rapid Acquisition with Relaxation Enhancement (RARE) sequence with VTR^24^ was used to generate T_1_ maps of the bladder wall before and after transurethral 0.3 mL instillation of an aqueous contrast mixture (CM) containing 4 mM Gadobutrol (Gadavist; Bayer, Wayne, NJ, USA) and 5 mM Ferumoxytol (AMAG Pharmaceuticals Inc., Waltham, MA, USA) via a 24-gauge angiocatheter (Becton–Dickinson Infusion Therapy Systems, Sandy, UT, USA). Imaging parameters were as follows: TR = 400, 842, 1,410, 2,208, 3,554 and 10,000 ms, echo time (TE) = 7 ms, 9 contiguous 0.7 mm-axial slices, RARE factor = 2, 2 signal averages, 20 × 20 mm field of view (FOV) and a matrix size of 200 × 200, zero-filled to 400 × 400 prior to Fourier transform. T_1_ maps were processed using a 3-parameter single exponential function.

### T_1_ measurement after protamine sulfate (PS) exposure for permeability validation

Following post contrast MRI, we first expelled the CM by pressing on the bladder followed by two transurethral irrigation of saline 0.5 mL prior to the instillation of 0.5 mL of PS (Sigma-Aldrich, St. Louis, MO, USA) 1% w/v in normal saline. After a dwell time of 30 min, PS was expelled from the bladder for a second instillation of CM (0.3 mL) for a repeat post-contrast T_1_ mapping.

### Real-time RT-PCR and histology

WAS and control group rats not exposed to PS were euthanized, the bladder harvested for either isolating RNA or histology after 30 min fixing with 4% paraformaldehyde (Sigma-Aldrich, St. Louis, MO, USA) at 4 °C, followed by overnight immersion in 20% sucrose at 4 °C for cryoprotection in optimal cutting temperature (OCT) preservation medium (Tissue-Tek, Torrance, CA, USA) before cryosectioning. Sections were stained with Hematoxylin and Eosin (Fisher Healthcare, Pittsburgh, USA) and then digital photographs were acquired. Researchers blinded to the identity of sections examined the regions of interest at low and high magnification for visible damage to the mucosal layer, edema, infiltration of polymorphonuclear inflammatory cells, and vascular congestion. The sections of two groups were subjectively scored as follows: a unit score was assigned for the evidence of mucosal thinning, another unit score for the evidence of vascular congestion in same area and likewise for the infiltration of inflammatory cells. All the scores were added to arrive at a composite score for the inflammation of each section from control and WAS groups when viewed at 40 × magnification.

Mucosa of freshly harvested bladder was separated^30,31^ and frozen immediately at − 80 °C until isolation of total RNA using TRizol reagent (Invitrogen, Carlsbad, CA, USA) for real-time PCR using primers and cycle conditions as previously reported^32^ for relative quantity of tight junction ZO-1 transcript normalized to GAPDH mRNA. The contamination of lamina propria cannot be excluded by our technique of manual separation of mucosa from detrusor. Primer sequences for ZO-1 (Forward 5′-GCGAGGCATCGTTCCTAATAAG-3′; Reverse 5′-TCGCCACCTGCTGTCTTTG-3′ and GAPDH forward primer 5′-AGACAGCCGCATCTTCTTGT-3′; Reverse 5′-GATACGGCCAAATCCGTTC-3′ were procured from Integrated DNA technologies, Coralville, IA, USA.

### Statistical analysis

All values are expressed as mean ± SD. Since T_1_ relaxation times are normally distributed, two-way analysis of variance (ANOVA) followed by Sidak’s multigroup comparison or unpaired *t* test for pairwise comparison of physiological parameters and Mann–Whitney test for the semi-quantitative parameter of inflammation scores using GraphPad Prism ver 8.0.0 (GraphPad Software, San Diego, CA, USA). Values at *p* < 0.05 were considered significant.

## Results

### Colonic motility index of psychological stress

When exposed to daily WAS for an hour over 10 days, rats in the WAS group expelled a significantly higher number of fecal pellets (4.54 ± 0.29 pellets/hour vs 1.08 ± 0.11 pellets/hour, *p* < 0.0001) compared to the control group.

### Metabolic cage

Rats in the WAS group voided significantly smaller single volumes in nocturnal void of 0.35 ± 0.04 mL compared to 0.76 ± 0.11 mL in the control group (*p* < 0.05) without any significant difference in the micturitions recorded over 12 h time-frame (15 ± 3.32/12 h vs 13.5 ± 3.76/12 h). Total urine volume was also significantly decreased in WAS rats (11.7 ± 6.25 mL vs 4.7 ± 2.24 mL; p < 0.05) (Table 1).

**Table 1:** Metabolic cage urination parameters.

Voiding parameter	Control	WAS	*p* value	95% confidence interval
Single Void Volume (mL)	0.76 ± 0.11	0.35 ± 0.04	< 0.05	− 0.6526 to − 0.1706
Number of micturition in 12 h	13.5 ± 3.76	15 ± 3.32	> 0.05	− 10.08 to 7.085
Urine output (mL)	11.7 ± 6.25	4.7 ± 2.24	< 0.05	− 13.79 to − 0.06559
Voiding Interval (min)	58.0 ± 23.2	60.6 ± 22.3	> 0.05	− 31.92 to 36.96

All values are expressed as mean ± SD.

### Voxel-wise T_1_ mapping

**Figure 1:**
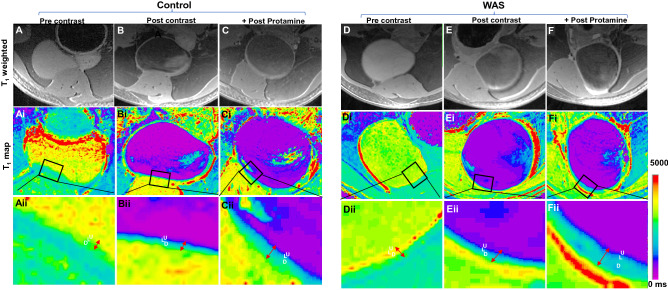
Effect of WAS and PS on voxel-wise T_1_ mapping- T_1_ weighted images (TR/TE 1410/7ms) acquired before the instillation of CM (pre-contrast), after the instillation of CM but prior to the exposure to protamine sulfate PS (post-contrast) and after PS exposure in the rat bladder from control (**A**–**C**) and WAS group (**D**–**F**). To derive the functional meaning of a slight increase in the signal intensity of WAS bladder wall, color coded, voxel-wise T_1_ maps with volumetric coverage were constructed from the mono exponential fit of signal in T_1_ weighted images acquired at six different TR values of 400–10,000 ms using aRARE factor of 2 and signal averages of 2. Just as the traditional histochemical stains reveal the biochemical differences between tissue layers, the color-coded T_1_ maps with the resolution of 50 µm for each pixel after zero filling displays the three individual layers of mucosa (U), lamina propria (L) and detrusor (D) in bladder wall differentiated by the physical parameter of T_1_ relaxation time mapped to voxels sized 0.1 × 0.1 × 0.7 mm^3^. The relative ingress of extracellular T_1_ shortening agent, Gadobutrol in the post-contrast T_1_ maps expanded the spatial separation between pixels displaying the layers of U and L (**Eii** vs **Bii**) and predicted an expansion of extracellular space in L of WAS group compared to controls. The spatial separation between U and L of WAS group increased further after a brief PS exposure (**Fii** vs **Cii**).

First a series of raw T_1_ weighted images were acquired at TR of 400, 842, 1410, 2208, 3554 and 10,000 ms while keeping TE constant at 7 ms to reduce the component of T_2_ weighting in the acquired images. For illustration, raw T_1_ weighted image taken at TR of 1410 ms is shown for each group in each experimental setting in Fig. 1A–F, which were acquired before the instillation of CM (pre-contrast), after 0.3 mL instillation of CM but prior to the exposure to protamine sulfate PS (post-contrast) and after PS exposure(post-protamine). In order to reconstruct the geographical representation of true T_1_ for different bladder wall layers within the FOV of 20 × 20 mm—voxel-wise T_1_ mapping—T_1_ was calculated from nonlinear least square data fitting of TR dependent signal intensity increase in every voxel of multiple raw T_1_ weighted images acquired at different TR of 400, 842, 1410, 2208, 3554 and 10,000 ms. The color-coded T_1_ maps (Fig. 1Ai–Fii) display purple and red color for T_1_ of 0 and 7000 ms, respectively.

To avoid the variability from bladder distension during imaging, urine production by the animals was reduced with 12 h water restriction prior to imaging and the representative T_1_ weighted images for the control (Fig. 1A–C) and WAS group (Fig. 1D–F) displays that the bladder distension was not variable across groups.

**Figure 2:**
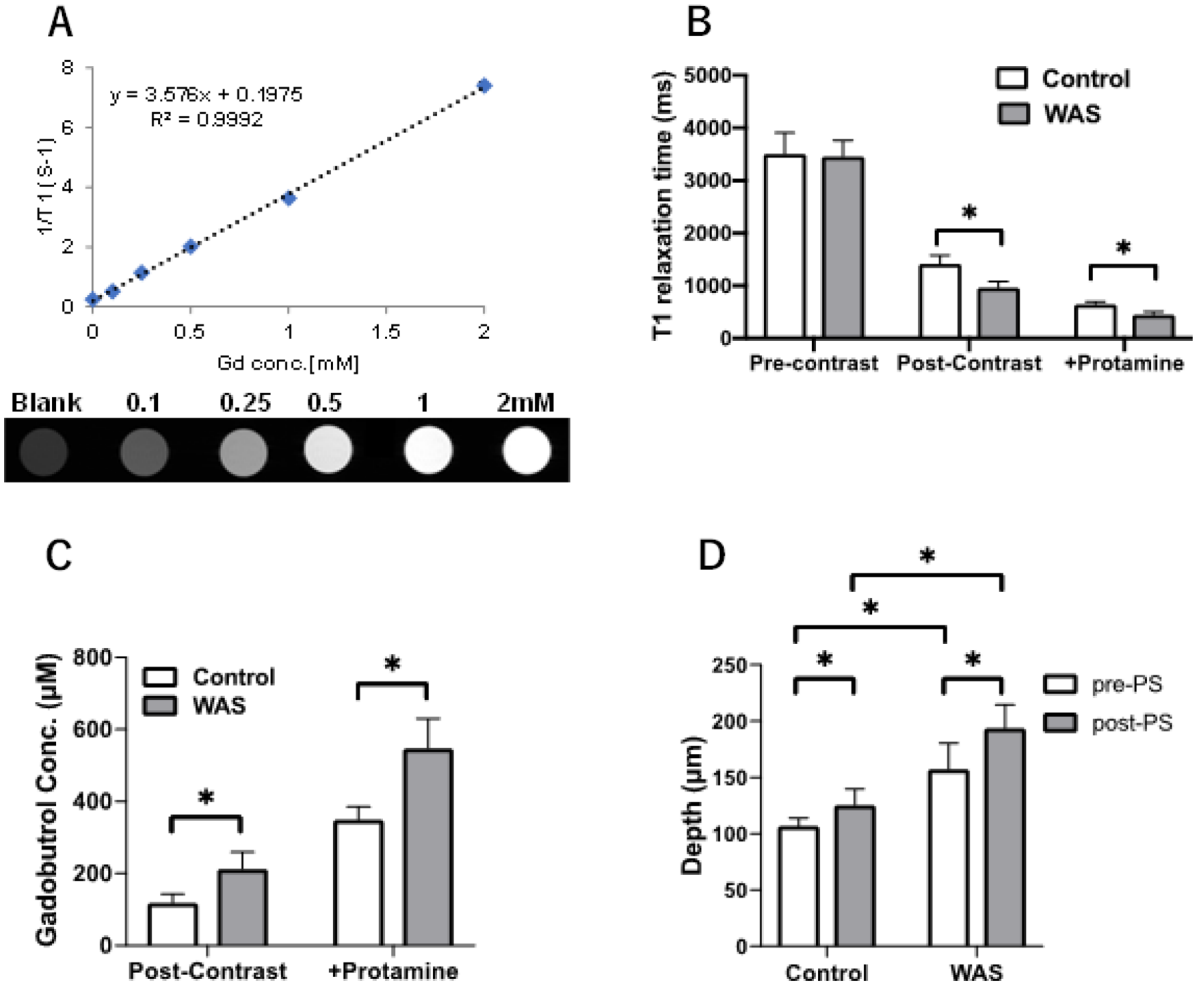
Gadobutrol Permeability Derived from Voxel-wise T_1_ mapping: (**A**) To derive the Gadobutrol concentration [Gd] in the bladder mucosa using the Eq. (1), we first estimated the T_1_ relaxation rate constant or relaxivity (*r*_1_) of Gadobutrol at 9.4 T by the linear fitting of the reciprocal of the water T_1_ relaxation time or T_1_ water relaxation rate (1/T_1_) measured in the serial two fold dilutions of [Gd] 2 mM at 37 °C. Linear fitting generated the regression equation: y = 3.576x + 0.1975 with the coefficient of determination (R^2^) of 0.999 and *r*_1_ value of 3.576 L/mmol/s for Gadobutrol at 9.4 T. In contrast to the linear relationship between T_1_ water relaxation rate of the phosphate buffered saline (PBS) vial (blank) supplemented with ascending concentrations of Gadobutrol from 0.1-2 mM [Gd], the non-linearity between [Gd] and signal intensity is illustrated by the doubling of [Gd] from 1 to 2 mM producing only a minor change in the signal intensity of T_1_ weighted images acquired at TR/TE of 640/14 ms. (**B**) Compared to control group, the post-contrast T_1_ relaxation time computed from 20 pixels in ROI of mucosa* was significantly shorter in WAS group (*p < 0.005, two-way ANOVA followed by Sidak’s test) and T_1_ relaxation time was further shortened upon PS exposure (0.5 mL of 1%w/v) for 30 min. (**C**) Higher mucosal permeability in WAS group is indexed by a significantly higher ingress of Gadobutrol derived from the non-invasive, quantitative measurement of increased T_1_ water relaxation rates (n = 5; *p < 0.001, two-way ANOVA and Sidak’s test) which is doubled from the respective pre-PS levels by PS exposure in both groups. (**D**) Bladder wall depth of instilled Gadobutrol penetration was evaluated by measuring the spatial separation between U and L on post-contrast and post-protamine T_1_ maps. The penetration depth of Gadobutrol after PS in WAS group was significantly larger than in the control group *p < 0.05. All values are expressed as Mean ± SD.

We compared the pre and post contrast T_1_ for 20 pixels in an ROI of the bladder wall highlighted by the square boxes in panels Ai-Fi-magnified in the respective lower panels (Aii–Fii) to visualize the sandwich of lamina propria (L) between the mucosa (U) and detrusor (D) layers. Given the pixel size for display is 50 µm in Fig. 1, at least two pixels display individual cells^33^ of the luminal cell layer of mucosa. The pre-contrast T_1_ values for the U (3509 ± 359 ms vs 3460 ± 280 ms; Fig. 2B), for L (3176 ± 144 ms vs 3058 ± 274 ms) and D (2632 ± 175 ms vs 2743 ± 84 ms) layers of control and WAS groups respectively, were comparable (Fig. 1Aii,Dii). After the instillation of CM in lumen, the long T_1_ of mucosa (Fig. 1Aii,Dii) is shortened to 1420 ± 144 ms and 965 ± 111 ms (Fig. 2B) in post-contrast T_1_ maps of control and WAS group, respectively (Fig. 1Bii,Eii) and the mean values of each group in different experimental settings were used for calculating the mean values of Gadobutrol permeability in each group.

### Gadobutrol permeability

As displayed in Fig. 1Ai-Di, the mucosa of WAS group exhibits significantly shorter post-contrast T_1_ relaxation time than that of controls (Fig. 2B) and the between group differences were further accentuated upon PS exposure (Fig. 2B). The shortening of T_1_ relaxation time can help us derive Gadobutrol permeability in any given region of interest (ROI) of bladder wall. Because the longitudinal relaxation rate of water (R1 = 1/T_1_) is linearly related to the Gadobutrol concentration^21,23,29^, Gadobutrol permeability can be computed from the pre-contrast and post-contrast T_1_ maps using the Eq. (1)

1 $$\Delta \left( \frac{1}{T1} \right) = \frac{1}{{ T1\left( {post} \right)}} - \frac{1}{{ T1 \left( {pre} \right)}} = r1\left[ {Gd} \right]$$

where [Gd] is the unknown Gadobutrol concentration, *r*_1_ is the relaxivity of Gadobutrol (measured 3.58 mM^−1^ s^−1^ at 9.4 T, Fig. 2A), and 1/T1(pre) and 1/T1(post) are the pre- and post-contrast spin–lattice relaxation rates derived from pre-contrast and post-contrast T_1_ relaxation time of respective groups (Fig. 2B).

With the help of Eq. (1), we calculated significantly higher [Gd] in the mucosa (U) of WAS group compared to controls (Fig. 2C). Using the threshold of ΔT_1_ > 1500 ms, deeper Gd penetration was measured in the WAS group 152.04 ± 17.8 µm vs 106.9 ± 5.81 µm in controls and greater still after PS exposure 193.92 ± 16.7 µm vs 125.55 ± 9.72 µm (Fig. 2D), PS is well known to evoke urothelial denudation^25^.

### Validation of MRI permeability assay

The robustness of MRI to assay the bladder permeability of WAS model is evident from the differential impact of PS exposure on the mucosal permeability of control and WAS groups (Fig. 2C). A significant increase in the ingress of Gd (~ 200 μM) in the WAS group upon PS exposure relative to controls (Fig. 2D) suggests that the stress aggravates the innate response to mild noxious stimulation in bladder, which can be relevant in understanding the flare-up of symptoms in IC/BPS symptoms.

### Histological confirmation

**Figure 3:**
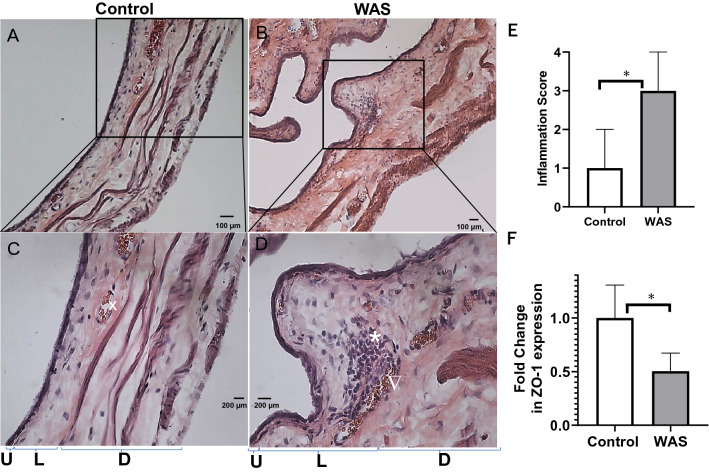
MRI successfully visualizes the sandwich of lamina propria (L) layer between the tissue layers of mucosa (U) and detrusor (D) and spatial separation of layers on MRI predicted the histological differences noted on H&E staining of rat bladder harvested prior to PS exposure from both groups. Compared to the histology of control rats (**A**–**C**), tissue sections from WAS group (**B**–**D**) exhibited a relative thinning of mucosa (U) with the decrease in the number of cell layers, expansion of lamina propria (L) from edema as evident from the relative expansion of L and D layers of WAS group compared to the control group (bladder wall thickening), focal areas of mononuclear inflammatory cells (*), venous congestion and elongated blood vessels (telangiectasia ∇) together supporting a prominent role for inflammation in WAS group even without PS exposure (**D**). (**E**) A significant elevation in the median semi-quantitative score of inflammation in WAS group relative to the control group (plot of median and interquartile range as error bars, n = 4, *p < 0.0001, Mann–Whitney Test) reflects the conspicuous absence of inflammatory cells in control group (**A**–**C**), a circular shape (x) instead of elongated blood vessels and mucosa with multiple layers and thinner bladder wall signifying a relative absence of edema compared to WAS group (**B**–**D**). (**F**) WAS exposure evokes barrier dysfunction per se even in the absence of PS exposure as evident from histological analysis and the thinning of mucosal layers is corroborated by the downregulation of tight junction protein ZO-1 in the mucosa of WAS group. Values are Mean ± SD; *p < 0.05, unpaired t test.

The visual separation of U and L layers predicted by the post-contrast MRI of the WAS group (Fig. 1Eii) was subsequently confirmed by the H&E staining of animals (n = 4) not exposed to PS (Fig. 3A–D). Compared to the low and high magnification images of control rat bladder (Fig. 3A,C) the images of WAS group without PS exposure (Fig. 3B,D) exhibited a thinned mucosa (U) with a decrease in the number of cell layers.

The extent of edema in WAS group as a consequence of inflammation led to a physical expansion of (L) layer as visible in Fig. 3D indicating that compared to controls, bladder wall thickening in WAS group occurs in conjunction with the multiple foci of mononuclear inflammatory cells (*), and venous congestion (∇) and elongated blood vessels (telangiectasia). The histology of bladder sections from control group was conspicuous by the absence of inflammatory cells. The median score of WAS group was significantly elevated compared to the control group with the statistical significance of non-normal data analyzed by Mann–Whitney test (*p* < 0.0001; Fig. 3E).

### Real-time PCR

The ZO-1 mRNA was downregulated in the bladder mucosa of WAS group compared to controls (0.50 ± 0.15 vs 1.00 ± 0.28, *p* < 0.05) (Fig. 3F).

## Discussion

To bring a paradigm shift in the permeability assay of bladder mucosa, we report on the measurement of bladder wall T_1_ relaxivity for indexing the innate host response to chronic psychological stress and PS exposure. Since bladder wall T_1_ relaxivity is linearly related to the paracellular entry of [Gd] into the extracellular space of bladder wall either across the tight junction of capillaries after intravenous injection of Gadobutrol or its analog)^24^ or across the tight junction complex of umbrella cells after instillation (as in irradiated mouse bladder^29^), we hypothesized that the quantitative T_1_ mapping of rat bladder can serve as a robust index for compromised bladder permeability of rat exposed to WAS^13^. A minimally invasive MRI based permeability assay can provide global information of bladder permeability and avoid the site-selection bias of biopsy^2,4,5,12^ or the complexity and errors intrinsic to biochemical assays^10,16^.

It is well known that the value of any physical parameter depends on the externally applied fields: e.g.*,* just as the weight of an object increases in the higher gravitational field of Earth vs Moon, T_1_ relaxation time of human bladder also ascends from 0.765 s, 0.923 s to 1.544 s with the ascending magnetic field strength of 0.35 T^34^, 1.5 T^24^ and 3 T^35^. Indeed, the histological changes noted on the harvested rat bladder with the chemical binding of histochemical stains were predicted by the voxel-wise T_1_ mapping and a deeper Gadobutrol diffusion reflected an expansion of extracellular space^23^ in lamina propria of the WAS group, subsequently confirmed by histology. We inferred that the significantly higher ingress of [Gd] in the WAS group is in concert with the histologically confirmed thinning of the urothelium and tight junction protein, ZO-1 downregulation in separated mucosa. The functional(MRI), histologic and molecular evidence for a barrier dysfunction in WAS is corroborated by several reports on barrier dysfunction^6–8^ including the dilatation of intercellular spaces in the urothelium of female WAS rat^13^ and the report of increased shedding of urothelium in female stressed rats^9^. When our findings are taken together with the reported downregulation of ZO-1 and E-cadherin^5,12^ in the bladder biopsy of BPS/IC patients^5,10,11^, we inferred that the exposure to acute or chronic psychological stress can precipitate an acute rise in bladder permeability which can exacerbate flare-up^36^ and/or worsen BPS/IC symptoms^1^.

It is well established that PS exposure increases the bladder permeability of rodent^25–27^ and of human bladder^16^ and therefore the doubling of respective Gadobutrol ingress after PS exposure from pre- PS exposure levels in both control and WAS groups validates our MRI based mucosal permeability assay. After PS exposure, the Gadobutrol levels in bladder of WAS group were computed to be ~ 0.5 mM comparable to the maximum plasma levels measured 2 min after the intravenous injection of Gadobutrol^37,38^. Furthermore, the doubling of instilled Gadobutrol ingress mirrors the doubling of radiolabeled urea ingress into rabbit^27^ and human bladder^16^ after PS exposure. Moreover, the mucosal permeability barrier of live animal is critically dependent upon the oxygenation via capillary perfusion^39,40^ as illustrated by the ten-fold uptake of radiolabeled urea after PS exposure ex vivo^26^ compared to just two-fold increase in vivo^27^*.* Infact, the differences in bladder mucosal permeability ex vivo^26^ and in vivo^27^ could be easily assessed by the percentage of non-viable cells ex vivo^20,41^.

Since mucosal concentration build up via diffusion tends to quickly reach an equilibrium with the luminal concentration of probe, the mucosal concentration of instilled probe was found to be largely independent of the instillation period in prior computer simulation^42^ of distributed model of intravesical pharmacokinetics as well as in empirical studies^43^. This occurs because the instilled drug diffusing into capillary perfused bladder wall^44^ gets cleared away by the venous blood to erect a downhill concentration gradient for sustaining the paracellular diffusion into mucosa and obviate any concentration buildup in the mucosa as observed ex vivo^26^. Therefore, the lack of any additional rise in the [Gd] ingress with the increase in the instillation period of CM for repeat T_1_ mapping after PS exposure demonstrates the compliance of [Gd] ingress with the distributed model for intravesical pharmacokinetics. The extensive plasma uptake^16,27^ of the instilled radiolabeled urea in IC/BPS patients^10^ through a first order process validated the distributed model for intravesical pharmacokinetics. The venous clearance of instilled Gadobutrol is argued by the venous clearance of the eight times larger inulin when instilled into rat bladder^44^ and therefore the venous clearance of diffused Gadobutrol not only engenders a logarithmic concentration decline across the bladder wall thickness^29^ but also prevent any additional rise in the mucosal concentration with the repeat instillation.

The passive, concentration dependent, paracellular diffusion of instilled Gadobutrol^29^(604.71 Daltons), cations^10^ and of large molecular weight permeability probes such Evans blue dye (961 Daltons)^17,45^ into bladder mucosa led us to infer that the compromised mucosal barrier increases the penetration of irritants from urine for inciting the underlying afferent nerves and drive the pain and LUTS of IC/BPS patients. Thus, a significant rise in [Gd] ingress after PS exposure in the WAS group can potentially replicate the flare up of BPS/IC symptoms^46^ in a non-infective rodent model without relying on bacterial products.

Instead of instilling permeability probe to 80% of functional capacity in controls and BPS/IC patients^15^, we choose to use a constant instilled volume of 0.3 mL for CM in both groups to exclude the role of bladder distension in observed permeability differences. Moreover, 0.3 mL is < 50% of the average nocturnal bladder capacity of awake control rats and much lower than the threshold of 90% anaesthetized bladder capacity required for inducing any alterations in the rabbit mucosal permeability^20^. While WAS model recapitulates the correlation of smaller bladder capacity with the bladder wall inflammation of BPS/IC patients^4^, we failed to detect any increase in the urinary frequency presumably due to variable urine production during night-time or due to a decrease in total urine volume of WAS group. The psychological stress evoked by unpredictable stressors is known to affect the endocrine release of corticosterone and adrenaline, which can cause colon motility, polydipsia and sleep disturbances^47^. Thus, the higher number of fecal pellets measured during 1 h WAS exposure supports our claim of psychological stress playing a critical role in this model, but the diurnal and nocturnal variation in water intake of the WAS group must be clarified in future studies. In addition, the effect of WAS on sleep cycle needs to be addressed for fully comprehending the effect of WAS on mucosal hyperpermeability.

Since bladder permeability is a key pathophysiological mechanism, at least in a subset of BPS/IC patients^1–5,10,11,16^, our clinically viable, pain free approach of T_1_ mapping together with a minimally invasive, instillation of CM at constant volume holds relevance  in potential phenotyping of BPS/IC patients into bladder-centric or extra-bladder phenotypes^4^. Instead of measuring the blood levels of instilled probes^15^ or urine levels^16,27^ to indirectly determine the levels of instilled probe taken up by bladder mucosa, MRI allows a direct, non-invasive measurement of probe levels in bladder mucosa and excludes the impact of large interindividual variations in the volume of distribution parameter^48^. It is likely that the failed clinical translation^15^ of instilled radiolabeled permeability probe levels in blood may be linked to the compromised mucosal perfusion of IC/BPS patients^39^. An eight fold elevation of instilled salicylate in feline models of IC^49^ and a two-fold elevation of a radiolabeled probe (~ 4 times the molecular mass of salicylate) after acute bladder injury ( akin to PS exposure) in rabbits^15^ failed to translate in IC/BPS patients^15^ because of the differences in mucosal perfusion of two cohorts^39^. The compromised mucosal perfusion may be a two-edged sword which can not only aggravate urothelial hyperpermeability but also hinder in the venous clearance of a diffused permeability probe before it can be measured in isolated blood aliquots. Thus, the use of intravesical contrast MRI allows us to better understand the mechanistic relationship between psychological stress and bladder dysfunction for advancing the understanding of the phenotype-specific BPS/IC pathology.

## Conclusion

The findings highlight the translational relevance of a physical assay for bladder permeability using a validated model of psychologically stressed rodents together with PS exposure to mimic the flare up of BPS/IC patients. T_1_ weighted MRI with a minimally invasive procedure of CM instillation can evaluate the bladder mucosal permeability to advance the diagnosis and the clinical care of BPS/IC patients.

## Supplementary Information

Supplementary Information.
