# Supplementary material for: Functional and histologic imaging of urinary bladder wall after exposure to psychological stress and protamine sulfate
Source: Sci Rep. 2021 Sep 30;11:19440. doi: 10.1038/s41598-021-98504-9 (PMC8484474; doi:10.1038/s41598-021-98504-9)
Supplement: Supplementary file 1 — Supplementary Information. [file 41598_2021_98504_MOESM1_ESM.pdf]

Peeling of rat bladder mucosa

Freshly harvested bladder from the sacrificed rat is cut open by scissors and remnants of urine are washed away with chilled phosphate buffered saline (PBS) and the tissue is immediately placed in petri dish containing the chilled PBS on ice bucket. Then, we use two sets of surgical forceps to peel the mucosa, with one set, we grab the mucosal layer that extends out from one end of the cut-open bladder and the detrusor is grabbed by the other set of forceps. Both forceps are gradually moved in opposite directions for separation of mucosa. Mucosa of freshly harvested bladder was separated [30,31] and immediately immersed in RNAlater and kept at -80℃ until isolation of total RNA using TRizol reagent (Invitrogen, Carlsbad, CA, USA) for real-time PCR using primers and cycle conditions as previously reported [32] for relative quantity of tight junction ZO-1 transcript normalized to GAPDH mRNA. The contamination of lamina propria cannot be excluded by our technique of manual separation of mucosa from detrusor. Primer sequences for ZO-1 (Forward 5'-GCGAGGCATCGTTCCTAATAAG-3'; Reverse 5'-TCGCCACCTGCTGTCTTTG-3' and GAPDH forward primer 5'- AGACAGCCGCATCTTCTTGT-3'; Reverse 5'- GATACGGCCAAATCCGTTC-3' were procured from Integrated DNA technologies, Coralville, IA, USA.

Data file for Fig.2D

**Depth of Gadobutrol Penetration in microns**					
**Control**	**Pre-PS**	**Post PS**	**WAS**	**Pre-PS**	**Post PS**
control1	102.333	132	stress1	139	183
control2	113	121	stress2	160.6	224
control3	99	108.2	stress3	131.87	173.2
control4	104.42	141	stress5	186.2	205.2
control5	115.62		stress7	142.56	184.2
Mean	106.8746	125.55	mean	152.046	193.92
Std. dev	5.81181359	9.725568	Std. dev	17.82751	16.70872
